# Stabilization of the Highly Hydrophobic Membrane Protein, Cytochrome *bd* Oxidase, on Metallic Surfaces for Direct Electrochemical Studies

**DOI:** 10.3390/molecules25143240

**Published:** 2020-07-16

**Authors:** Anton Nikolaev, Iryna Makarchuk, Alexander Thesseling, Jo Hoeser, Thorsten Friedrich, Frédéric Melin, Petra Hellwig

**Affiliations:** 1 Laboratoire de Bioelectrochimie et Spectroscopie, UMR 7140, Chimie de la Matière Complexe, Université de Strasbourg, CNRS, 67081 Strasbourg, France; teterev3000@mail.ru (A.N.); makarchuk.iryna@etu.unistra.fr (I.M.); 2 Institut für Biochemie, Fakultät für Chemie und Pharmazie, Albert-Ludwigs-Universität Freiburg, 79104 Freiburg, Germany; thesseling@bio.chemie.uni-freiburg.de (A.T.); jo.hoeser@gmail.com (J.H.); friedrich@bio.chemie.uni-freiburg.de (T.F.)

**Keywords:** membrane protein, *bd* oxidase, lipids, self-assembled monolayer (SAM), electrocatalysis, thiols, gold nanoparticles (NP)

## Abstract

The cytochrome *bd* oxidase catalyzes the reduction of oxygen to water in bacteria and it is thus an interesting target for electrocatalytic studies and biosensor applications. The *bd* oxidase is completely embedded in the phospholipid membrane. In this study, the variation of the surface charge of thiol-modified gold nanoparticles, the length of the thiols and the other crucial parameters including optimal phospholipid content and type, have been performed, giving insight into the role of these factors for the optimal interaction and direct electron transfer of an integral membrane protein. Importantly, all three tested factors, the lipid type, the electrode surface charge and the thiol length mutually influenced the stability of films of the cytochrome *bd* oxidase. The best electrocatalytic responses were obtained on the neutral gold surface when the negatively charged phosphatidylglycerol (PG) was used and on the charged gold surface when the zwitterionic phosphatidylethanolamine (PE) was used. The advantages of the covalent binding of the membrane protein to the electrode surface over the non-covalent binding are also discussed.

## 1. Introduction

Membrane proteins are encountered in essential physiological processes such as transport, signaling, respiration and photosynthesis. It is estimated that in the next century 80 to 90% of “drugable” targets are membrane proteins [1]. Their contributions to the energy state of the cell are ruled by their conformation, position and reactivity of specific residues in response to the electrochemical ion gradients that are composed of an electrical and a concentration component. There is a large discrepancy between the significance and knowledge of membrane proteins that is linked to the difficulties associated with the handling and study of these proteins [2,3]. The interaction of membrane proteins with their “solvent”, the membrane lipids, is not well understood [4]. The functional properties of membrane proteins are modulated by specific interactions with lipids [5,6], but also by specific interactions with cytosolic proteins, adding a further level of complexity. Preserving the structure and function of the membrane protein after extracting them from their natural environment thus remains a challenge.

Here, we focus on cytochrome *bd* oxidase, a membrane protein found in the respiratory chains of bacteria, including several pathogens [7,8]. It is suggested to be involved in the protection against oxidative stress, as well as in the virulence, adaptability and resistance of these bacteria [9,10,11] and is thus a potential target for the discovery of novel antibiotics [12,13,14]. Cytochrome *bd* oxidase catalyzes the reduction of oxygen to water and couples this reaction to the oxidation of quinone. It contains three heme centers in the catalytic subunit (CydA), namely heme *d*, heme *b_558_* and heme *b_595_*. A hydrophilic loop (known as the “*Q*-loop”) connecting transmembrane helices 6 and 7 in CydA is believed to be involved in quinone binding [15,16,17,18]. Two structures of *bd* oxidase are now available, the X-Ray structure of the enzyme from *Geobacillus thermodenitrificans* [19] and the cryo-EM structures of the *bd*-*I* from *Escherichia coli* [20,21]. They clearly show that *bd* oxidases are highly hydrophobic and nearly completely embedded in the membrane.

The study of the oxygen reaction via direct electrochemistry on gold nanoparticles (NPs) modified with thiols was recently presented by our group, with focus on the influence of pH, protein concentration and quinone content [22]. The catalytic activity of the immobilized proteins was found optimal at pH 7 and for concentrations in the range of 0.6–1.0 mg/mL. The surface coverage of active protein on the electrode was then 0.1 pmol·cm^−1^, and their turnover rate (k_cat_) 100 s^−1^ ± 25%, as determined from a Koutecky–Levich plot [22]. The long-term protein film stability after immobilization was also investigated previously in our group. The response of the modified electrode with time showed that after two hours only 5% of the activity was lost, while after 120 h the enzyme still kept 50% of the catalytic signal. The observed loss in the catalytic current was likely due to partial enzyme desorption or deactivation of the membrane protein with time [22].

Here, we present a systematic analysis of the parameters that rule the stability of the protein films and electrocatalytic behavior after non-covalent immobilization the proteins, including lipid content and electrode surface charge. In addition, a comparison of the electrocatalytic properties of the proteins covalently immobilized to the electrode surface is also provided.

## 2. Results and Discussion

### 2.1. Identification of Protein Stability and Analysis of the Catalytic Response

**Figure 1:**
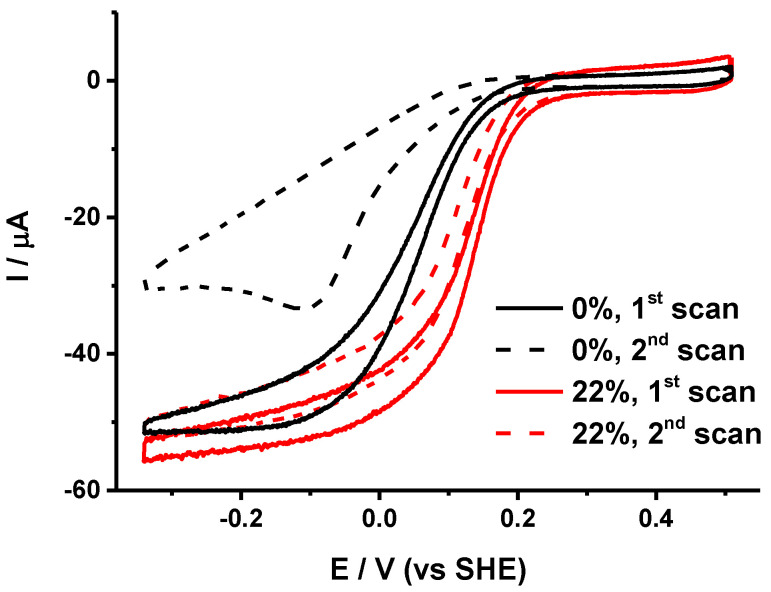
Two consecutive voltammograms obtained for partially delipidated cytochrome *bd* oxidase (black plots) and the same preparation reconstituted with 22% phosphatidylglycerol/ phosphatidylethanolamine (PG/PE) (1/1) (red plots) on gold nanoparticles (NPs) modified with HT/MCH (1/1).

In the presence of oxygen, cytochrome *bd* oxidase non-covalently immobilized on rotating electrodes modified with gold nanoparticles and thiols gave rise to quasi-sigmoidal curves which are typical for the electrocatalytic reduction of O_2_ (Figure 1). The analysis of the cyclic voltammograms (CV) was conducted utilizing the following parameters:

(i) The half wave potential E_cat_ of the sigmoidal curve, which is related to the kinetic efficiency of the oxygen reaction. The higher E_cat_, the lower the overpotential for O_2_ reduction.
(ii) The slope Δi/ΔE of the catalytic curve in the region of limiting current, which provides information on the distribution of the orientations of the proteins on the electrode surface [23].
(iii) The variability Δi/i of the limiting current value between two consecutive scans separated by 10 min that allows the evaluation of the stability of the protein films.

The above three parameters for each protein immobilization condition examined in the present study are collected in Table 1. The standard error of measurements (SEMs) were calculated from three different measurements.

### 2.2. Requirement of Lipids for Stabilization of the Protein Film

The sample with the lowest lipid amount was co-immobilized with 0, 5, 15, 22, 30, respectively, and 44% of a 1/1 mixture of PG and PE lipids on gold NPs modified with HT/MCH (1/1). These lipids are both encountered naturally in high amounts in the *E. coli* membranes [24]. Figure 1 shows the first two consecutive scans obtained for 0 and 22% lipid contents. Upon increasing the lipid content up to 22%, the stability of the electrochemical signal between two consecutive scans increased (see Table 1). For higher lipid contents, the stability eventually decreased. Thus, 22% seems to be the best lipid content for this preparation to get the most stable signal. Interestingly, for such a lipid amount the E_cat_ is also higher and the limiting current exhibits only a moderate slope, which suggests that the proteins are immobilized rather homogeneously on the electrode surface with a favorable orientation for electron transfer. Lipids are not only crucial to the structural integrity of cytochrome *bd* oxidase but may also directly influence the heme redox potentials and thus the catalytic mechanism of the enzyme [25,26].

### 2.3. Mutual Influence of Lipid Type and Electrode Surface Charge

**Figure 2:**
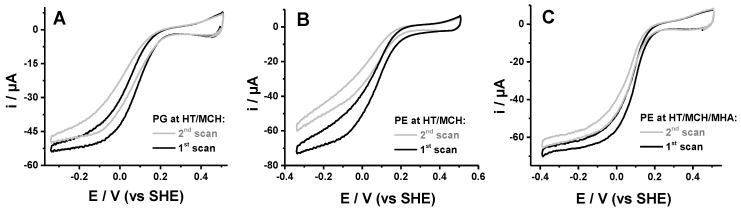
Two consecutive voltammograms obtained for cytochrome *bd* oxidase with 2.5% added PG on gold NPs modified with HT/MCH (1/1), (**A**) cytochrome *bd* oxidase with 2.5% added PE on gold NPs modified with HT/MCH (1/1) (**B**) and cytochrome *bd* oxidase with 2.5% added PE on gold NPs modified with HT/MCH/MHA (1/1/1) (**C**).

The influence of the lipid charge was then examined by varying the amounts of PG or PE in the films. The former lipid exhibits a negatively charged head group whereas the latter has a zwitterionic head group (when including the phosphate group). For this study, the second preparation containing more lipids was used. As compared to the partially delipidated one, this sample already led to protein films with higher stability on gold NPs modified with a mixture of neutral thiols HT and MCH. The addition of 2.5% PG slightly improved the catalytic response (see Table 1 and Figure 2A). Indeed, E_cat_ was upshifted by about 20 mV, and the protein films were more stable. In contrast, the addition of 2.5% PE led to a downshift of E_cat_, an increase in the slope of the limiting current, and a decrease in the film stability (Figure 2B). More realistic catalytic parameters were restored if some thiols with charged head groups were present on the gold surface. Interestingly, both MPA (negative charge) and cyst (positive charge) improved the catalytic response of cytochrome *bd* oxidase with 2.5% added PE. The most stable signal was obtained with MPA, whereas cyst led to the highest E_cat_. The lipid type and surface charge, therefore, act in synergy in the immobilization of cytochrome *bd* oxidase. The best catalytic responses are obtained on the neutral surface when negatively charged PG is used and on the charged surface when zwitterionic PE is used. The addition of lipids had no effect on the activity of the preparations, which was 3250 ± 300 (U/mg protein).

### 2.4. Influence of the Length of the Thiol

**Figure 3:**
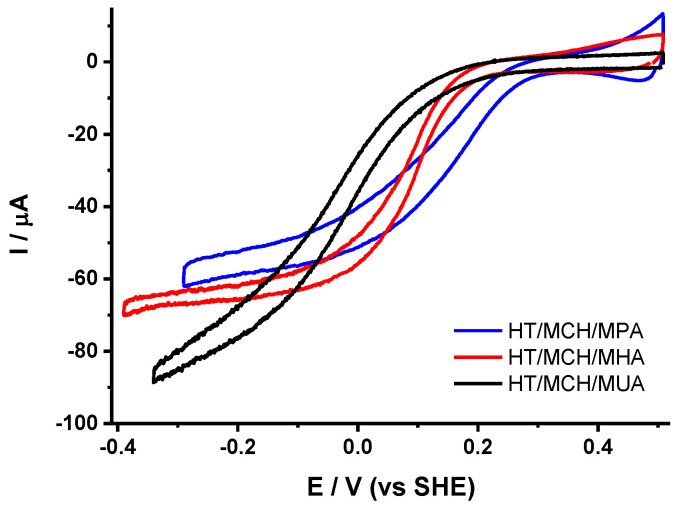
Voltammograms obtained for cytochrome *bd* oxidase with 2.5% added PE on gold NPs modified with HT/MCH/MPA (1/1/1) (blue trace), HT/MCH/MHA (1/1/1) (red trace) and HT/MCH/MUA (1/1/1) (black trace).

The influence of the chain length of the ω-carboxyl alkanethiol on the electrocatalytic properties of immobilized cytochrome *bd* oxidase with 2.5% added PE was also studied (see Figure 3). Upon increasing the chain length from 3 to 11 carbons, E_cat_ was significantly downshifted. This is probably due to a decrease in the interfacial electron transfer rate when the distance between the metallic surface and the protein increases, as discussed before for soluble model proteins [27,28,29,30]. Interestingly, the stability of the films also decreased at the same time, which suggests that the proteins are less tightly bound on the surface when longer thiols are used. The effect on the orientation of the proteins on the surface was less straightforward. The slope of the limiting current was higher for both MPA and MUA and lower in the case of MHA. Both short and long thiols led to a higher distribution of protein orientations on the electrode surface. For short thiols, an increased amount of defects in the self-assembled monolayer (SAM) [29] could be the origin of the observation.

### 2.5. Covalent vs. Non-Covalent Attachment of the Protein

**Figure 4:**
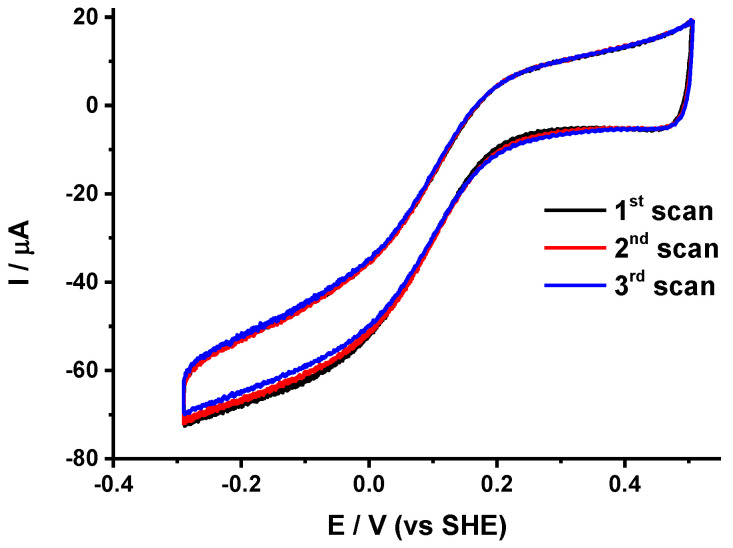
Three consecutive voltammograms obtained for cytochrome *bd* oxidase covalently immobilized on gold NPs modified with Ni-NTA self-assembled monolayers (SAMs).

For covalent attachment of the protein to the gold NPs surface, we have taken advantage of the high affinity of the hexahistidine tag (His-tag) genetically introduced for purification purposes on the surface of the protein for nickel (II) nitrilotriacetic acid (Ni-NTA) complex. This strategy was successfully used before for the immobilization of various membrane proteins, including photosystem I [31], cytochrome *c* oxidase [32], respiratory complex I [33] and glucose transporters [34]. The Ni-NTA self-assembled monolayer was prepared on the gold surface in three steps. The gold surface was first modified with a thiol containing an *O*-succinimide ester (DTSP), which was then coupled to Nα′,Nα″-bis(carboxymethyl)-l-lysine. In the last step, Ni^2+^ ions were added. The voltammograms obtained after immobilization of the protein are shown in Figure 4.

As anticipated, the stability of the protein films was much higher after covalent immobilization compared to simple immobilization on gold NPs modified with thiols. Figure 4 shows that only 2% of the catalytic signal was lost after three successive scans (see Table 1), which is the best stability achieved so far for cytochrome *bd* oxidase films. The small decrease in the signal can be due to slow deactivation of the protein over time. The mid-wave potential E_cat_ obtained here (0.12 V) was also in the range of the values obtained before for immobilization on thiols. Although a higher level of homogeneity in protein orientation was expected from covalent attachment through the His-tag, the slope of the limiting current (0.052 μA·mV^−1^) suggests that this is not the case. It is very likely that the attachment of the protein through the His-tag on the opposite side of the *Q*-loop and the hemes does not allow to efficiently control the electron transfer path, and thus does not prevent a dispersion of interfacial electron transfer rates. It is also noted that the presence of nickel ions trapped in the films leads to twice higher capacitive current values compared to previous modified electrodes. The Ni^2+^ ions probably form a double layer near the electrode surface that influences capacitance.

## 3. Materials and Methods

### 3.1. Chemicals

Sodium citrate, hydrogen tetrachloroaurate trihydrate, sulfuric acid, ubiquinone-1, 1-mercaptohexanol (MCH), 1-hexanethiol (HT), mercaptohexanoic acid (MHA), mercaptoundecanoic acid (MUA), 3,3′-dithiodipropionic acid di(*N*-hydroxysuccinimide ester) (DTSP), dimethyl sulfoxide (DMSO), Nα′,Nα″-bis(carboxymethyl)-l-lysine, and nickel sulfate were purchased from Sigma (St-Louis, MO, USA); potassium phosphate dibasic trihydrate from Acros Organics (Geel, Belgium); mercaptopropionic acid and cysteamine (cyst) from Tokyo Chemical Industry (Tokyo, Japan). The 1,2-dioleoyl-*sn*-glycero-3-phospho-*rac*-(1-glycerol) (sodium salt) (PG) and 1,2-dimyristoyl-*sn*-glycero-3-phosphoethanolamine (PE) were provided by Avanti Polar Lipids Inc. (Alabaster, AL, USA). These chemicals were used without any additional purification. Thiols were dissolved in ethanol, and lipids were prepared in a 1/3 (*v*/*v*) mixture of ethanol and chloroform.

### 3.2. Protein Preparation

Cytochrome *bd* oxidase from *E. coli* was purified as previously described [35]. The protein harbors a His-tag on the *C*-terminus of the catalytic subunit CydA, on the opposite side of the *Q*-loop and the hemes. The residual lipid content of the protein sample may vary in function of the preparation (detergent used, salt) and washing procedure. For illustration, we worked with two different protein samples. The first sample was washed more thoroughly than the second to remove loosely bound lipids. It is noted that in some membrane proteins, for example the *bc_1_* complex [36,37], it was found that some lipids cannot be removed and thus may be still present after washing. Prior to adsorption on the modified electrodes, the amount of detergent was also decreased by washing 5 µL of the protein sample solution with a potassium phosphate buffer (100 mM, pH 7) on a 50 kDa cutoff Amicon filter. Protein solutions of 10 µM final concentration gave the best results and were incubated for 1 h with the corresponding quantities of lipids and ubiquinone-1 in a glass vessel at 4 °C. The activity of the preparations was determined amperometrically using a Clark-type oxygen electrode (Hansatech, Oxygraph+, Hansatech, Pentney, UK) as previously described [38].

### 3.3. Electrode Modification for Non-Covalent Attachment of the Protein

After mechanical polishing with 0.3 μm aluminium oxide powder and electrochemical pretreatment in 0.1 M H_2_SO_4_, gold disk electrodes (4.5 mm diameter) were modified with three successive 9 µL depositions with the solution of 15 nm gold NPs. The gold NPs were prepared by the procedure of Turkevich et al. [39] and Frens [40]. Briefly, a solution of HAuCl_4_ (49 mg) in 125 mL of water was boiled, and subsequently a solution of sodium citrate (143 mg in 13 mL water) was added. The solution was kept under boiling temperature for 15 min, cooled down to room temperature and concentrated by centrifugation at 10,000 rpm during 30 min and removal of 95% of the supernatant. The gold surface was then modified overnight with 1 mM mixtures of either HT/MCH, HT/MCH/MPA, HT/MCH/MHA, HT/MCH/MUA or HT/MCH/cyst in EtOH. After rinsing with fresh ETOH and drying, 6 μL of protein solution were deposited on the surface of the electrode and kept at 4 °C for 3 h. The protein excess was removed by rinsing with a 100 mM pH 7 phosphate buffer solution.

### 3.4. Electrode Modification for Covalent Attachment of the Protein

The deposition of gold NPs on the electrode surface was carried out as described above, but this time the gold surface was modified for 1 h with a 2 mM solution of DTSP in DMSO. The excess DTSP was then rinsed away with fresh DMSO and the electrode surface was dried under an argon stream. Next, it was covered with 100 mM Nα′,Nα″-bis(carboxymethyl)-l-lysine in 0.5 M K_2_CO_3_ at pH 9.8 for 3 h and afterwards washed with water. Finally, the surface was incubated with 50 mM NiSO_4_ for 1 h before rinsing again with water. Then 5 µL of a 10 µM protein solution were deposited on the modified electrode surface and kept at 4 °C for 1 h. The protein excess was removed by rinsing with a 100 mM pH 7 phosphate buffer.

### 3.5. Electrochemical Measurements

Measurements of the background and protein electrochemical activity were conducted in a 100 mM pH 7 phosphate buffer with a conventional three electrode cell connected to a potentiostat (Versastat 4, Princeton Applied Research (Ametek), Oak Ridge, TN, USA). Silver chloride electrode (3 M KCl) was used as the reference electrode and a platinum wire as the counter electrode. All the potentials mentioned here are referenced versus the standard hydrogen electrode. All the voltammograms shown have been recorded under air at 20 °C with 0.02 V·s^−1^ scan rate and 1000 rpm electrode rotating speed, which gives a good compromise between signal intensity and the perturbations from the rotation. For each modification, the experiment was repeated three times to assess the reproducibility.

## 4. Conclusions

The lipid type and electrode surface charge mutually influenced the stability of the films of non-covalently immobilized cytochrome *bd* oxidase. The best electrocatalytic responses were obtained on the neutral gold surface when a negatively charged PG was used and on the charged gold surface when zwitterionic PE was used. The stability and electrocatalytic performance were also influenced by the length of the thiols. Short thiols offered both a better stability and lower overpotential but led to a higher distribution of protein orientations on the metal surface. Interestingly, the covalent immobilization of the protein through the His-tag significantly increased the stability of the films but did not lead to a higher level of homogeneity in protein orientation. The stability of the protein on the electrode surface is important for future studies for the identification of inhibitors from small molecule libraries. We note, however, that since the inhibitors have to access the quinone binding site, the parameters for protein orientation are further tuned for the screening.

## Figures and Tables

**Table 1:** Half wave catalytic potential (E_cat_), slope of the limiting current (Δi/ΔE) and variability of the limiting current value between two consecutive scans separated by 10 min (Δi/i), obtained for the different immobilization conditions tested in this study. Standard error of measurements (SEMs) are 0.02 V for E_cat_, 0.003 A·mV^−1^ for Δi/ΔE and 0.02 for Δi/i.

Immobilization Conditions				E_cat_	Δi/ΔE	Δi/i
Sample	% Lipid	Lipid Type	Thiol Type	(V)	(μA·mV^−1^)	
1	0	-	HT/MCH (1/1)	0.06	0.007	0.39
1	5	PE/PG (1/1)	HT/MCH (1/1)	0.16	0.017	0.26
1	15	PE/PG (1/1)	HT/MCH (1/1)	0.16	0.020	0.13
1	22	PE/PG (1/1)	HT/MCH (1/1)	0.16	0.008	0.07
1	30	PE/PG (1/1)	HT/MCH (1/1)	0.16	0.020	0.08
1	44	PE/PG (1/1)	HT/MCH (1/1)	0.11	0.009	0.40
2	0	-	HT/MCH (1/1)	0.11	0.013	0.09
2	2.5	PG	HT/MCH (1/1)	0.13	0.012	0.06
2	2.5	PE	HT/MCH (1/1)	0.08	0.052	0.15
2	2.5	PE	HT/MCH/MPA (1/1/1)	0.16	0.028	0.04
2	2.5	PE	HT/MCH/MHA (1/1/1)	0.11	0.010	0.07
2	2.5	PE	HT/MCH/MUA (1/1/1)	0.01	0.023	0.10
2	2.5	PE	HT/MCH/cyst (1/1/1)	0.20	0.019	0.08
2	2.5	PE	Ni-NTA	0.12	0.052	0.02
